# Time tO last ChemotherApy and death in ovaRian cancEr patients: TO CARE/MITO 42 study, a retrospective analysis of italian MITO centers

**DOI:** 10.3389/fonc.2025.1641758

**Published:** 2025-08-06

**Authors:** Giulia Scotto, Anna Galatà, Robert Fruscio, Giulia Besana, Fabio Landoni, Luca Sgro, Alessandra Testi, Fulvio Borella, Gennaro Cormio, Mariangela Gianciotta, Francesca Arezzo, Brigida Anna Maiorano, Alessandra Baldoni, Marinella Destefanis, Jole Ventriglia, Sandro Pignata, Rita Chiari, Maria Carmen Azzolina, Ivano Raimondo, Margherita Turinetto, Valentina Tuninetti, Massimo Di Maio, Giorgio Valabrega

**Affiliations:** ^1^ Medical Oncology, ASL TO3 Ospedale degli Infermi, Rivoli, Torino, Italy; ^2^ Department of Oncology, University of Turin, Turin, Italy; ^3^ UO Gynecology, Fondazione IRCCS San Gerardo, Monza, Italy; ^4^ Department of Medicine and Surgery, University of Milan-Bicocca, Milan, Italy; ^5^ Obstetrics and Gynaecology Unit, Umberto I Hospital, Department of Surgical Sciences, School of Medicine, University of Turin, Turin, Italy; ^6^ Gynecology and Obstetrics Unit 1, Department of Surgical Sciences, City of Health and Science University Hospital, University of Turin, Turin, Italy; ^7^ Gynecologic Oncology Unit, IRCCS Istituto Tumori “Giovanni Paolo II”, Bari, Italy; ^8^ Interdisciplinar Department of Medicine, University of Bari “Aldo Moro”, Bari, Italy; ^9^ Department of Precision and Regenerative Medicine - DiMePRe-J, University of Bari “Aldo Moro”, Bari, Italy; ^10^ Department of Medical Oncology, IRCCS San Raffaele Hospital, Milan, Italy; ^11^ Oncology and Hematology Department, Mirano AULSS3 Serenissima, Mirano, Italy; ^12^ Dipartimento Chirurgico ASO S. Croce e Carle Cuneo, Cuneo, Italy; ^13^ Department of Urology and Gynecology, Istituto Nazionale Tumori IRCCS - Fondazione G. Pascale, Naples, Italy; ^14^ UOC ONCOLOGIA AST Pesaro Urbino, Pesaro Urbino, Italy; ^15^ AO Ordine Mauriziano, Torino, Italy; ^16^ School in Biomedical Sciences, University of Sassari, Sassari, Italy; ^17^ Department of Gynecology, Mater Olbia Hospital, Olbia, Italy; ^18^ Department of Oncology, University of Turin, Medical Oncology, Ordine Mauriziano Hospital, Turin, Italy; ^19^ Department of Oncology, University of Turin, AOU Città della Salute e della Scienza di Torino, Turin, Italy

**Keywords:** ovarian cancer, chemotherapy, end of life, quality of life, gynecologic cancer

## Abstract

**Introduction:**

The European Society for Medical Oncology (ESMO) 2021 Guidelines contraindicate the administration of chemotherapy in the last month of patients’ life. The main objective of this multicenter observational retrospective study was to calculate the time elapsed between the date of the last chemotherapy and the date of death of patients with ovarian cancer. The secondary objectives were to identify any factors associated with a greater probability of receiving chemotherapy in the end of life.

**Methods:**

Ovarian cancer patients operated between 2010 and 2020 in the participant Italian centers were enrolled. Only deceased patients whose date of death and date of last chemotherapy were known were included.

**Results:**

603 women from 10 Italian centers were included. One patient out of four (25.7%) received chemotherapy in the last month of life. The median survival from the last chemotherapy was 66 days. Patients with a neutrophil/lymphocyte ratio ≥5, with high C-reactive protein at the start of the last line and patients dying in hospital compared to hospice/palliative care at home were more likely to undergo chemotherapy at the end of life (p<0.001, p=0.05 and p<0.001 respectively). Being treated in Northern Italy reduces the chance of receiving chemotherapy at the end of life in comparison with Center-South (p<0.001), as well as being enrolled in at least one clinical protocol (p=0.027).

**Discussion:**

The TO CARE/MITO 42 study is a snapshot of the Italian practice in which there are still disparities in the treatment of patients at the end of life. A prospective observational study could provide useful elements for early identification of patients who would not benefit from a further line.

## Introduction

Ovarian cancer is the eight cancer for incidence and at the fifth place for mortality in Europe in 2022 (1). The prognosis, although improved in recent years, remains poor, especially in advanced stages, and most of the patients diagnosed with Fédération Internationale de Gynécologie et d’Obstétrique (FIGO) stage III or IV tumors will develop recurrence within 18 months (2, 3). At the fifth recurrence, survival is around five months (4). The term ‘end of life’ is frequently used but poorly defined (5). The European Society for Medical Oncology (ESMO) Guideline refers to ‘end of life’, for people with advanced disease, as ‘point of rapid physical decline, typically the last few weeks or months before an inevitable death as a natural result of a disease’ (6). ESMO Guidelines explicitly contraindicate chemotherapy and immunotherapy in the last weeks of life (6). Chemotherapy in the last month of life is associated with adverse outcomes including poor quality of care, emergency department attendance, cardiopulmonary resuscitation, mechanical ventilation and dying in an intensive care unit. These items are defined as indicators of aggressive end of life care by several authors in the literature (7, 8).

Despite these indications, there are large retrospective registry-based American studies showing aggressive care in the final months of life in patients with ovarian cancer, including chemotherapy administration, emergency room access, hospital and intensive care unit admissions (9–14). Other retrospective and registry-based trials conducted in different countries around the world have shown a similar scenario of aggressive treatment at the end of life (15–17). In contrast, there is little data on the European reality on this topic. A large Dutch registry-based study has been published in recent years: of the 1775 patients included, only 12% received chemotherapy in the last month of life (18). The only data on the Italian scenario come from the EOLO study, a retrospective single-center Italian study, that included 110 patients dead of advanced/recurrent ovarian cancer and 38% of these had chemotherapy during the last month of life (19). The cut-off of chemotherapy administration at the end of life varies between studies (9–19). 30 days is the one most used regarding ovarian carcinoma (6, 9, 12, 16, 18–21).

Several parameters representing systemic inflammatory responses have been reported to be prognostic parameters in patients with ovarian cancer. Among these the neutrophil/lymphocyte ratio has been described as negative prognostic factor in ovarian cancer, as well as in other tumor types (22). Furthermore, in a study of patients with recurrent ovarian cancer, higher neutrophil/lymphocyte ratio was identified as prognostic factors for mortality within 100 days of failure of last line of chemotherapy (23).

The modified Glasgow Prognostic Score, which is classified as 0, 1, or 2 calculated by combining C-reactive protein and albumin values, has also been identified as an independent predictor of overall survival in patients with recurrent ovarian cancer (24). Also, C-reactive protein value has been described in literature as negative prognostic parameter in advanced cancer and in ovarian cancer (16, 25) The aim of this study is to describe the Italian reality about chemotherapy in the end of life in a cohort of ovarian cancer patients, and to identify factors associated with the probability of receiving chemotherapy in this period.

## Materials and methods

### Patient selection and data collection

TO CARE/MITO 42 is an Italian multicenter retrospective observational study. Data analysis was conducted by collecting the medical records of ovarian cancer patients treated at the oncology Day Hospitals (Oncology and Gynecologic Oncology) of the Multicenter Italian Trials in Ovarian cancer and gynecologic malignancies (MITO) Centers participating in Italy.

Patients who underwent surgery for ovarian carcinoma between January 2010 and December 2020 in participating MITO centers were selected. In addition, only deceased patients whose date of death was known and whose date of last chemotherapy treatment performed was known were included.

The following general data were then retrospectively recorded: patient characteristics, pathological data (histotype, grading), FIGO stage at diagnosis, first line treatment, subsequent treatment, BRCA status and inclusion or non-inclusion in clinical protocols. Regarding the focus of the study, clinical and laboratory data were collected before the start of the last line of chemotherapy, drugs used, end of life place, activation or not of home palliative care services at the start of the last therapy, date of administration of the last chemotherapy, date of death. Patients who had hormone therapy as the last line were described, but the date of last chemotherapy considered referred to the previous line.

After approval by the Ethics Committee of the Coordinating Center Hospital Umberto I, Mauriziano in Turin, the encrypted database and study protocol was sent to all Principal Investigators of the 160 MITO centers in Italy. Participating centers have obtained approval from local Ethics Committees.

### Statistical analysis

Given the exploratory nature of this retrospective analysis, based on the collection of cases available at participating centers, the sample size is not based on a predefined hypothesis. It was estimated to collect about 600 patients before conducting the analysis. The sample estimate was made on a survey we sent to the MITO centers in which they provided us with indicative numbers of patients they would include. The data analysis was descriptive. Continuous variables were described by median, interquartile range and absolute range. Categorical variables were described by frequency and percentage. The main analysis was based on the dichotomous endpoint “chemotherapy in the last 30 days of life” (yes vs no). The proportion of patients who received chemotherapy in the last 30 days in the overall case series and subgroups, identified based on potential factors impacting this outcome (age, performance status, number of previous lines, end of life place), also selected on the basis of literature data, was described.

The association of individual factors was tested with contingency tables and chi-square tests. Multivariate logistic regression analysis was performed, including clinical variables statistically significant (p<0.05) at univariate analysis. Laboratory variables were not included in the multivariate model due to the high number of patients with data not available.

Post-cancer treatment survival was calculated as the time from the date of last chemotherapy administration to the date of death. Consistent with the study’s inclusion criteria, which limited the analysis to deceased patients, there were no censored patients.

The Kaplan-Meier method was used to calculate survival after the last chemotherapy treatment. Analyses with p value < 0.05 were considered significant. Due to exploratory nature of the study, no adjustments were made for the multiplicity of statistical tests.

## Results

A total of 603 patients from 10 MITO centers participating (response rate 6%, 10/160 contacted centers) were analyzed (**Table 1**).

**Table 1:** Participating centers and included patients.

Center	Included patients	Percentage
MAURIZIANO	103	17
AORMN	10	1.7
CSS-SGR	49	8
IRCCS S GERARDO MONZA	188	31.2
MATER OLBIA HOSPITAL	1	0.2
MIRANO	23	3.8
NAPLES	10	1.7
POLICLINICO BARI	98	16.3
S.ANNA	100	16.6
VERDUNO	21	3.5
**TOTAL**	**603**	**100**

AORMN, Azienda Ospedali Riuniti Marche Nord; CSS-SGR, Casa Sollievo dalla Sofferenza S. Giovanni Rotondo; IRCCS, Istituto di Ricovero e Cura a Carattere Scientifico.

### Descriptive analysis

**Figure 1:**
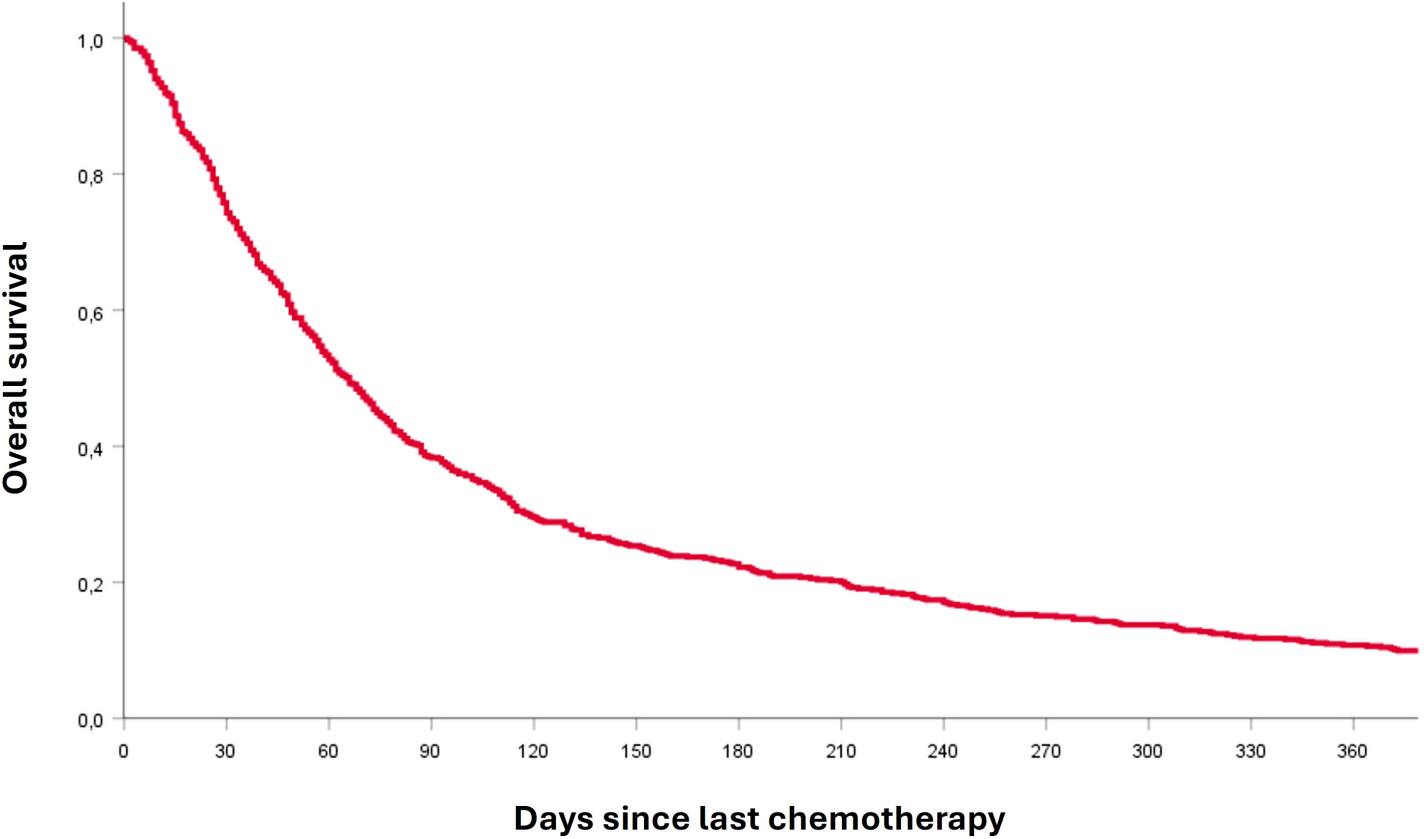
Kaplan-Meier survival curve from the day of the last chemotherapy administration to death.

The characteristics of patients are shown in **Table 2**. Median age at diagnosis was 64 years (range 27-89). Most patients had serous histotype ovarian cancer (76.1%) and high-grade tumor (78.9%). In addition, almost 90% of patients had advanced FIGO stage (64.3% had FIGO stage 3 and 25.5% had FIGO stage 4). Among 320 patients who were tested for BRCA1/2 status, 78 (24%) had a mutation; this information is missing in 283 patients. For 77 patients (12.8%) the last active treatment was weekly paclitaxel; for 76 patients (12.6%) pegylated liposomal doxorubicin; for 68 patients (11.3%) carboplatin-paclitaxel; 62 patients (10.3%) had gemcitabine as last line of chemotherapy; 51 patients (8.5%) had hormone therapy; 45 patients (7.5%) carboplatin monotherapy; 43 patients (7.1%) metronomic oral cyclophosphamide; 35 patients (5.8%) carboplatin-pegylated liposomal doxorubicin. Fewer patients (<5%) received carboplatin-gemcitabine, cisplatin, carboplatin-paclitaxel-bevacizumab, pegylated liposomal doxorubicin-trabectedin, topotecan, etoposide, gemcitabine-vinorelbine, carboplatin-gemcitabine-bevacizumab, vinorelbine, oxaliplatin, and epirubicin as last line, while 1% of patients have performed therapy within clinical trials. Median number of chemotherapy lines was 3 (range; 1-9). Namely, 344 patients (57%) received less than 3 lines of chemotherapy while 259 patients (43%) received more than 3 lines. Chemotherapy in the last 30 days of life was administered to 155 patients (25.7%). Median overall survival from the last chemotherapy administration was 66 days (Confidence Interval (CI) 95%, 59-73). The Kaplan-Meier survival curve from the day of last chemotherapy administration to death is shown in **Figure 1**.

**Table 2:** Patients’ characteristics.

Patients’ characteristics	
**Patients, n.**	603
**Median age at diagnosis, years**	64 (27 – 89)
Histotype, n (%)	
Serous	459 (76.1%)
Mucinous	17 (2.8%)
Endometrioid	30 (5%)
Clear cells	25 (4.1%)
Undifferentiated	26 (4.3%)
Mixed	42 (7%)
Grade, n (%)	
G1	11 (1.8%)
G2	54 (9%)
G3	476 (78.9%)
G4	15 (2.5%)
FIGO Stage at diagnosis, n (%)	
I	24 (4%)
II	23 (3.8%)
III	388 (64.3%)
IV	154 (25.5%)
BRCA 1/2 status, n (%)	
Mutate	78 (12.9%)
Wild Type	242 (40.1%)
Missed	283 (46.9%)
Number of lines, n (%)	
Less than or equal 3 lines	344 (57%)
More than 3 lines	259 (43%)
**Median number of lines, n (IQ range)**	3 (1-9)

PLD, Pegylated liposomal doxorubicin; FIGO, Fédération Internationale de Gynécologie et d'Obstétrique; BRCA 1/2, BReast CAncer 1/2.

### Analysis of association

The association of various factors with the likelihood of chemotherapy administration in the last 30 days of life was analyzed (**Table 3**). End-of-life place (palliative care at home/hospice vs. hospital) was significantly associated with the administration of chemotherapy (p<0.001) in the last 30 days: only 19.8% of patients whose end of life place was palliative care at home/hospice received chemotherapy, while 41.4% of patients who died in hospital received chemotherapy in the last thirty days. Enrollment in at least one clinical trial during the disease history was found to be significantly associated (p=0.027) with receiving chemotherapy at the end of life: patients enrolled in at least one protocol had a lower probability (16.1%) compared to non-enrolled patients (27.3%). Having a neutrophil/lymphocyte ratio greater than or equal to 5 at the start of last therapy was associated with a higher likelihood of receiving chemotherapy in end-of-life than having a neutrophil/lymphocyte ratio lower than 5 (40.6% vs. 22.6%, p<0.001). Borderline significance was found for the C-reactive protein, which was tested at the start of the last chemotherapy in only 139 patients (23.1%): it was observed that 27.6% of patients with a C-reactive protein ≥5 received chemotherapy in the last 30 days of life, while 12.2% of patients with a C-reactive protein <5 received therapy (p=0.05). Finally, the association between centers of origin (North/Central-South) and chemotherapy administration at the end of life was statistically significant: patients afferent to a center in the North were less likely to receive chemotherapy in the last thirty days than patients in the Central-Southern Italy (19.5% vs. 41.7%, p<0.001).

**Table 3:** Relationship between variables and probability of receiving chemotherapy in the end of life.

Variables		Chemotherapy in the last 30 days		p-value
		Yes	No	
Age	≥70	56 (22.9%)	189 (77.1%)	0.19
	<70	99 (27.7%)	259 (72.3%)	
Performance Status	PS 0-1	62 (28.3%)	157 (71.7%)	0.34
	PS ≥2	92 (24.7%)	280 (75.3%)	
End of life place	Hospice/palliative care at home	46 (19.8%)	186 (80.2%)	<0.001
	Hospital	41 (41.4%)	58 (58.6%)	
Enrollment in at least one clinical trial	Yes	14 (16.1%)	73 (83.9%)	0.027
	No	141 (27.3%)	375 (72.7%)	
Neutrophil/lymphocyte ratio	≥5	52 (40.6%)	76 (59.4%)	<0.001
	<5	82 (22.6%)	281 (77.4%)	
C-reactive protein	≥5	27 (27.6%)	71 (72.4%)	0.050
	<5	5 (12.2%)	36 (87.8%)	
Period of treatment	2010-2013	23 (30.7%)	52 (69.3%)	0.77
	2014-2016	32 (25.2%)	95 (74.8%)	
	2017-2020	54 (24.5%)	166 (75.5%)	
	2021-2023	46 (25.4%)	135 (74.6%)	
Number of chemotherapy lines	≤3	92 (26.7%)	252 (73.3%)	0.50
	>3	63 (24.3%)	196 (75.7%)	
Modified Glasgow Prognostic Score	≥2	9 (26.5%)	25 (73.5%)	0.25
	<2	12 (16.9%)	59 (83.1%)	
BMI	≤ 18.5	96 (26.5%)	266 (73.5%)	0.59
	>18.5	7 (31.8%)	15 (68.2%)	
Palliative care at home activation at last chemotherapy	Yes	61 (22.6%)	209 (77.4%)	0.12
	No	94 (28.2%)	239 (71.8%)	
Centers of origin	North	85 (19.5%)	350 (80.5%)	<0.001
	Central/South	70 (41.7%)	98 (58.3%)	

BMI, Body Mass Index.

At multivariate logistic regression analysis, including clinical variables with a statistically significant association at univariate analysis, 2 clinical variables included were significantly associated with chemotherapy administration at the end of life. In detail, chemotherapy administration at the end of life was less likely in patients enrolled in at least 1 clinical trial (Odds Ratio (OR) 0.43, 95% CI 0.20 – 0.95, p=0.037) and was more likely in patients treated in centers from Central-Southern Italy (OR 3.60, 95% CI 1.94 – 6.69, p<0.001). In addition, chemotherapy administration at the end of life was more likely in patients whose end of life place was the hospital compared to home/hospice (OR 1.73, 95% CI 0.96 – 2.14, p=0.07), although the result was not statistically significant.

## Discussion

The TO CARE/MITO 42 is the first multicenter study that analyzed the Italian practice about chemotherapy in the end of life in ovarian cancer patients. Our case series had 603 patients, from ten Italian centers, 5 centers in Northern Italy and 5 centers in Central and Southern Italy, all belonging to the MITO group, a cooperative research group in the field of gynecologic oncology. The fact that we chose only centers belonging to the MITO group allowed us to select a homogeneous case series, both from the surgical and oncological treatment point of view and reasonably representative of the Italian landscape. About one patient out of four (25.7%, 155/603) received chemotherapy in the last thirty days of life, in contrast to ESMO Guidelines 2021 (6), highlighting how this aggressive treatment at the end of life is still to be considered a very complex and under-studied topic.

The neutrophil/lymphocyte ratio is reported to be a prognostic factor in ovarian cancer, as well as in other tumor types (23). In a meta-analysis including nearly 3000 patients from 10 trials, patients with high pre-treatment neutrophil/lymphocyte ratio were found to have worse overall survival and progression free survival than patients with low pre-treatment neutrophil/lymphocyte ratio (22). In our case series, this subgroup of patients with neutrophil/lymphocyte ratio ≥5 at last-line initiation, using the cut-off used in the study by Roncolato et al. (20), in addition to having worse prognosis, in agreement with the literature, also showed to be more likely to receive, improperly, chemotherapy at the end of life (p<0.001). Thus, this is a selection of worse prognosis patients, potentially at risk of death in the short term, as also shown in the study by Kiuchi et al. (21)

Elevated C-reactive protein is also considered a negative prognostic factor for ovarian cancer patients (25, 26). We observed how patients with elevated C-reactive protein (cut-off of 5 mg/dL) at the start of the last line of chemotherapy seem to be more likely to receive chemotherapy at the end of life (p=0.05). This result is borderline statistical significance; however, it should be considered that this data was only available for 139 patients (23.1% of the total case series), reducing the statistical power of the analysis. In the study by Utsumi et al. (16) a lower performance status, high white blood cell count and high C-reactive protein were significantly correlated with shorter survival after the last treatment performed (p=0.004, 0.006 and 0.027, respectively). These observations spur us to perform complete blood tests on patients before starting a new line of treatment, including, of course, complete blood count but also laboratory parameters indicative of systemic inflammation, such as, indeed, C-reactive protein.

Death in hospital is among factors considered from the National Quality Forum as an indicator of aggressive medical care (27). In our case series, patients who died in the hospital (99 patients), compared with patients who died having activated palliative care at home or admitted to hospice (232 patients), were more likely to be treated with chemotherapy at the end of life (p<0.001). This result also agrees well with the literature, as shown by studies by Kajiyama and colleagues conducted in Japan, albeit on a smaller case series (28, 29). Recognizing when a patient can benefit from early activation of palliative care even during active treatment, is crucial and can make the transition between active treatment and best supportive care less difficult and sharp, avoiding unnecessary and harmful treatments for the patient, but also costly for public health (10).

The association between the geographical distribution of the center where the patient was treated (North vs. Center-South of Italy) and chemotherapy administration in the last 30 days is interesting: only 19.5% of patients enrolled in centers in the North received chemotherapy at the end of life, compared with 41.7% of patients from the Center-South (p<0.001). There are objective and documented differences in palliative care services between Northern and Southern Italy, as well as different management of resources in the territory (30). It should also be noted that, in our case series, the majority of patients enrolled in centers in the north were from university hospitals or research centers compared to those enrolled in the south (n=391 from university centers in the north versus n=108 from the south). This imbalance in source centers could further explain the difference found in the management of these patients, as clinicians working at university hospitals or research centers may have more experience in treating patients at the end of life.

These data urge us to have a more in-depth discussion among colleagues and, in the context of a multicenter group such as MITO, to look for ways to improve and try to increasingly smooth out such differences between centers. Patient enrollment within clinical protocols is a significant finding, but in contrast with already published data in the literature (31). In fact, being enrolled in at least one clinical protocol during disease history would lead to a lower likelihood of chemotherapy at the end of life (p=0.027). Certainly, the experience of “investigator” clinicians in MITO centers may have given reason for how they are able, especially for patients enrolled in research trials, to recognize when it is necessary to stop with active treatments. It is important to emphasize that these conclusions should be applied with caution to first-line patients. In fact, an outcome benefit has also been described for ovarian cancer patients who, despite their low performance status (Performance Status Eastern Cooperative Oncology Group ≥3), initiate first-line chemotherapy (32).

The retrospective nature of the study is a weakness and, in addition, the analyzed case series suffers from missing data. On the other hand, there is no Italian work of this magnitude in the literature (more than 600 patients). Our case series also included 63 patients with an interval from last chemotherapy to death of more than one year. It is more plausible that these patients did not die from ovarian cancer, although the retrospective nature of the case history does not allow us to be certain, as ‘cause of death’ data was not collected. However, we consider that since this population represents only 10% of all potential patient, this data did not impair the main analysis.

### Recommendations for clinical practice and future research

This Italian multicenter retrospective study showed that about one quarter of ovarian cancer patients who underwent surgery between 2010 and 2020 received chemotherapy in the last thirty days of life. The TO CARE/MITO 42 trial has effectively outlined the Italian practice regarding the topic of chemotherapy at the end of life. These data should encourage the development of standardized national policies for end-of-life care and the design of prognostic tools for the timely integration of palliative care. In addition, it becomes necessary to explore interventions to reduce regional disparities. Prospective observational clinical trials are needed to create tools for clinicians to identify women with a particularly poor prognosis who are unlikely to benefit from chemotherapy in order to avoid unnecessary, expensive, and toxic treatments in the last days of life.

## Data Availability

The raw data supporting the conclusions of this article will be made available by the authors, without undue reservation.
